# Editorial: The role of the microbiota-gut-brain axis in the pathogenesis of neurodegenerative diseases

**DOI:** 10.3389/fnins.2024.1393840

**Published:** 2024-04-02

**Authors:** Daniele Lana, Giada Magni, Maria Grazia Giovannini

**Affiliations:** ^1^Department of Health Sciences, Section of Clinical Pharmacology and Oncology, University of Florence, Florence, Italy; ^2^Institute of Applied Physics “Nello Carrara”, National Research Council (IFAC-CNR), Florence, Italy

**Keywords:** gut microbiota, Alzheimer's disease, neurodegenerative diseases, neurodegeneration, neuroinflammation

The microbiota is considered a unique integrated system with the gut (MB-gut). The brain is in continuous dialogue with the MB-gut, forming the MB-gut-brain axis. MB products activate several bottom-to-top pathways, necessary for the correct development and functionality of the central nervous system. Dysbiosis can affect normal brain aging, increasing neurodegenerative disorders, therefore the study of the interrelations of MB-gut with immune cells, neurons and glia is crucial to elucidate the molecular basis of neuronal diseases. The analysis of the neuron-glia alterations driven by the MB-gut will highlight the effects of dysbiosis, which, differentially recruiting and activating glia cells, may increase neurodegenerative mechanisms. Once defined the interrelation between MB-gut and glia, it will be of the utmost importance to elucidate which MB-gut alterations could be targeted to prevent, delay or decrease neurodegeneration. The present Editorial introduces the new Research Topic published on Frontiers in Neurosciences: “*The role of the microbiota-gut-brain axis in the pathogenesis of neurodegenerative diseases*” that covers this important topic of scientific research with a collection of six contributions, three Original Research Articles, and three Reviews.

In their original research article Zhang et al. studied the effects of probiotics on neurocognitive problems associated with perioperative gut dysbiosis and the involvement in this mechanism of NLRP3 pathway. Authors investigated learning and memory and inflammatory responses in mice undergoing surgery and in those administered with cefazolin, FOS + probiotics and CY-09. Surgery, or surgery/anesthesia decreased the animal freezing behavior but cefazolin attenuated this effect. Probiotics ameliorated memory impairments and postoperative memory deficits induced by perioperative cefazolin 3 weeks after surgery. NLRP3 levels increased 1 week after surgery and were attenuated by CY-09 and probiotics. In conclusion, authors showed that probiotics could correct dysbiosis and inflammatory response. Thus, probiotics seem an efficient way for maintaining the balance of gut microbiota, reducing NLRP3-related inflammation and alleviating postoperative neurocognitive disorders.

The research from Corley et al. investigated the effects of cyclophosphamide, methotrexate, and fluorouracil (CMF), a drug combination used to treat breast cancer, on depressive-like behavior in adult female mice and their effects on MB-gut. CMF induced social and despair-like behavior, modifying various proteins related to working memory impairments and anxiety disorder, identified by proteomics. Gene expression showed that NMDA and AMPA receptors increased in the hippocampus and amygdala of treated mice. Authors also observed CMF induced immediate changes in the microbial population. In conclusion, Corley et al. showed that cognitive changes associated to chemotherapy that occur in tandem with changes in the intestine and microbiome are the results of the modulation of the MB gut-brain axis.

In their original research article Medeiros et al. investigated the effect of a probiotic diet for 12 weeks on dysbiosis in a 3x-Transgenic-AD mouse model and explored its effects on disease progression.

Behavioral tests demonstrated improvements in memory performance in probiotic-fed AD mice. Neural tissue analysis of the entorhinal cortex and hippocampus of 10-month-old AD mice demonstrated that astrocytic and microglia densities were reduced in probiotic-fed AD mice. Furthermore, elevated numbers of neurons in the hippocampus suggested neuroprotection induced by probiotic supplementation. Results from this research suggest that probiotic supplementation could be effective in delaying or mitigating early stages of neurodegeneration in AD mice and that probiotic supplementation could provide an inexpensive and easily implemented adjuvant clinical treatment for AD.

In their review Ullah et al. presented recent updates on MB-gut role in the physiology and pathology of the organism. They showed that the communication between CNS and MB-gut includes chemical, neural immune and endocrine routes and that the alterations in the MB-gut can cause different disorders of the gastrointestinal tract. Dysbiosis leads to changes in MB-gut interplay and in the CNS, which are linked to neurological disorders pathogenesis. Many preclinical and clinical researches reported by authors indicate that gut microbiota changes are susceptibility factor for neurological disorders progression, including multiple sclerosis (MS), Alzheimer's disease (AD), Parkinson's disease (PD), and autism spectrum disorder (ASD). In their review, Ullah et al. discuss the crucial importance of the connection between MG-gut with the brain, the signaling pathways of peripheral and central biological systems and the contribution of MB-gut to many neurological disorders.

The review from Sun et al. is focused on the role of extracellular vesicles (EVs) as cell-to-cell and inter-organ communicators. Authors showed that gut bacterial EVs have a pivotal role in the MB-gut-brain axis and elicit distinct signaling to the brain, exerting regulatory function on neurons and glia. Authors showed that gut bacterial EVs with probiotic properties confer distinct therapeutic effects in various neurological disorders, suggesting that they may be a cause and a therapy for neuropathological disorders. Authors illustrated the basic, clinical, and translational studies on the therapeutic potential of gut microbial Evs for the treatment of neurological diseases, including Alzheimer's and Parkinson's disease, stroke and dementia. The review describes recent studies focused on the development of superior therapeutic microbial EVs using genetic manipulations and dietary interventions.

In the review from Liang et al. Alzheimer's disease emerges as a devastating neurodegenerative pathology in which the MB-gut assumes a cardinal significance. Authors presented recent investigations that illustrate the marked differences of the gut microbiota composition between AD patients and healthy people. Liang et al. explained that the configuration of the MB-gut has a participatory role in the initiation and progression of AD, in the complex milieu of the gut-brain axis. Authors focalized on the mechanistic role exerted by the enteric microbiota upon AD and illustrated therapeutic strategies that may form the bedrock of AD management.

In conclusion, the contributions of this Research Topic focus their attention on the importance of the interplay among MB-gut, immune cells, neurons, and glia. Authors clearly showed the implications of this communication for host defense, tissue repair and neurodegeneration. Modulation of MB-gut-brain axis may be a novel and viable strategy for the prevention and treatment of many neurodegenerative disorders.

## Author contributions

DL: Writing – original draft, Writing – review & editing. GM: Writing – review & editing. MG: Writing – original draft, Writing – review & editing.

